# The relativistic causality versus no-signaling paradigm for multi-party correlations

**DOI:** 10.1038/s41467-019-09505-2

**Published:** 2019-04-12

**Authors:** Paweł Horodecki, Ravishankar Ramanathan

**Affiliations:** 1 International Centre for Theory of Quantum Technologies, University of Gdańsk, Wita Stwosza 63, 80-308 Gdańsk, Poland; 2 Faculty of Applied Physics and Mathematics, National Quantum Information Centre, Gdańsk University of Technology, Gabriela Narutowicza 11/12, 80-233 Gdańsk, Poland; 3 Laboratoire d’Information Quantique, CP 224, Université Libre de Bruxelles (ULB), 1050 Brussels, Belgium; 4 Department of Computer Science, The University of Hong Kong, Pokfulam Road, Hong Kong, Hong Kong

## Abstract

The ubiquitous no-signaling constraints state that the probability distributions of outputs of any subset of parties in a Bell experiment are independent of remaining parties’ inputs. These constraints are considered to form ultimate limits for physical correlations and led to the fields of post-quantum cryptography, randomness generation besides identifying information-theoretic principles underlying quantum theory. Here we show that while these constraints are sufficient, they are not necessary to enforce relativistic causality in multi-party correlations, i.e., the rule that correlations do not allow casual loops. Depending on the space-time coordinates of the measurement events, causality only imposes a subset of no-signaling conditions. We first consider the n-party Bell experiment (*n* > 2) and identify all configurations where subsets of the constraints suffice. Secondly, we examine the implications for device-independent cryptography against an eavesdropper constrained only by relativity, detailing among other effects explicit attacks on well-known randomness amplification and key distribution protocols.

## Abstract (web summary)

It is generally assumed that no-signalling constraints and relativistic causality are equivalent. Here, the authors show that, in the multipartite setting, the no-signalling condition is in general stronger than demanding relativistic causality.

## Introduction

The recent experimental confirmation of the violation of Bell inequalities^1,2^ in systems of electron spins, entangled photons^3–5^, etc. has made a compelling case for the “non-locality” of quantum mechanics. Quantum phenomena exhibit correlations between space-like separated measurements that appear to be inconsistent with any local hidden variable explanation. The “spooky action at a distance” of quantum nonlocality is now embraced and utilized in fundamental applications, such as device-independent cryptography and randomness generation^6–8^ and reductions in communication complexity^9^. Moreover, this nonlocality has also been used to show that even a tiny amount of free randomness can be amplified^10,11^ and that extensions of quantum theory, which incorporate a particular notion of free choice, cannot have a better predictive power than quantum theory itself^12^. The quantum nonlocal correlations are known to be fully compatible with the no-signaling principle, i.e., the space-like separated parties cannot use the nonlocal correlations to communicate superluminally.

Since the proposal of Popescu and Rohrlich^13,14^, it has been realized that nonlocal correlations might take on a more fundamental aspect. Not only quantum theory but any future theory that might contain the quantum theory as an approximation is now expected to incorporate nonlocality as an essential intrinsic feature. This program has led to the formulation of device-independent information-theoretic principles^15,16^ that attempt to derive the set of quantum correlations from among all correlations obeying the no-signaling principle. In parallel, cryptographic protocols have been devised based on the input–output statistics in Bell tests, such that their proof of security only relies on the no-signaling principle. When one considers such post-quantum cryptography^6,7^, randomness amplification^10,11,17,18^, etc., the eavesdropper Eve is assumed to be limited to the preparation of boxes (input–output statistics) obeying a set of constraints collectively referred to as the no-signaling constraints. The general properties of no-signaling theories have been investigated^19^ in a related program to formulate an information-theoretic axiomatic framework for quantum theory. On the other hand, quantum theory does not provide a mechanism for the nonlocal correlations. Several theoretical proposals have been put forward to explain the phenomenon of nonlocal correlations between quantum particles via superluminal communication between them. These models go beyond quantum mechanics but reproduce the experimental statistical predictions of quantum mechanics, the most famous of these models being the de Broglie–Bohm pilot wave theory^20^.

In all relativistic theories, causality is imposed, i.e., the requirement that causes must precede effects in all space–time rest frames. Before going further, two remarks are in order here. First, by an effect, we mean any possible event, even if it has been affected by other events (causes) indirectly. Second, we shall use as general a correlation point of view as possible, *regardless* of the physical theory from which the correlations arise. In this context, let us also note that given an arbitrary space–time structure, the question of causal order for any two measures has been formalized using intuitions from optimal transport theory^21^. From the perspective of communication, the requirement of relativistic causality strictly demands that no faster-than-light (FTL) transmission of information takes place between a sender and a receiver. The no-signaling principle being in ubiquitous use (in device-independent cryptography, axiomatic formulations, etc., as explained earlier), a natural question is to explore whether the no-signaling constraints that are currently in use precisely capture the constraints imposed by relativistic causality, i.e., to derive the no-signaling constraints from relativistic causality.

In this paper, we investigate this question and find several surprising results outlined here. We initially establish the setup of the Bell experiment and recall the assumptions in the Bell theorem. We then define the notion of relativistic causality that we use in this paper (and that is commonly accepted, i.e., that there be no causal loops in space–time) and revisit the derivation of the two-party no-signaling constraints from causality. We find that in the two-party scenario, the usual no-signaling constraint requiring independence of any party’s output from the inputs of any space-like separated party exactly captures the notion of relativistic causality that causal loops are prohibited. We then show that, in the multiparty scenario, in certain space–time measurement configurations, only a restricted subset of the no-signaling constraints is required to ensure that no causal loops appear. We explicitly identify a region of space–time for the measurement events in a Bell scenario where the usual no-signaling constraints fail. In this regard, we extend a particular framework of “jamming” nonlocal correlations by Grunhaus, Popescu, and Rohrlich in ref. ^22^ based upon an earlier suggestion of Shimony in ref. ^23^. In particular, while they considered an infinite speed jamming mechanism for nonlocal correlations, we identify the entire region of space–time for all superluminal velocities of the point-to-region influences. We then examine the implications of the restricted subset of no-signaling constraints for device-independent cryptographic tasks against an eavesdropper constrained only by the laws of relativity. We detail explicit attacks on known protocols for randomness amplification based on the GHZ–Mermin inequalities using boxes that obey the new relativistic causality conditions. We show that from this perspective, the security theory needs revision. We also explore the implications on some of the known features of no-signaling theories^19^; in particular, we find that the phenomenon of monogamy of correlations is significantly weakened in the relativistically causal theories and that the monogamy of CHSH inequality violation^24^ disappears in certain space–time configurations. The notions of freedom of choice and no signaling are known to be intimately related^10^. We re-examine how the notion of free choice as proposed by Bell and formalized by Colbeck and Renner^25,26^ can be stated mathematically within the structure of a space–time configuration of measurement events. A breakthrough result in ref. ^27^ was a claim that any finite superluminal speed explanation of quantum correlations could lead to superluminal signaling and must hence be discarded. We re-examine this question in light of the modified relativistic causality and free-will conditions. Both nonrelativistic quantum theory and relativistic quantum field theory are well-known to obey a no superluminal signaling condition^28^, and proposals to modify quantum theory by introducing non-linearities have been shown to lead to signaling^29,30^. We end with discussion and open questions concerning the feasible mechanisms for the point-to-region superluminal influences.

## Results

### Notation

Let us first establish the notation for the typical Bell setup. In the Bell scenario denoted **B**(*n*, *m*, *k*), we have *n* space-like separated parties, each of whom chooses from among *m* possible measurement settings and obtains one of *k* possible outcomes. The inputs of the *i*th party will be denoted by a random variable (r.v.) *X*_*i*_ taking values *x*_*i*_ in [*m*] = {1, …, *m*} and the outputs of this party will be denoted by r.v. *A*_*i*_ takes values *a*_*i*_ in [*k*]. Accordingly, the conditional probability distribution of the outputs given the inputs will be denoted by

1 $$\begin{array}{l}P_{A_1, \ldots ,A_n|X_1, \ldots ,X_n}(a_1, \ldots ,a_n|x_1, \ldots ,x_n): = \\ P(A_1 = a_1, \ldots ,A_n = a_n|X_1 = x_1, \ldots ,X_n = x_n).\end{array}$$

Following refs. ^10,25^, we also consider the notion of a space–time random variable (SRV), which is a random variable *R* together with a set of space–time coordinates $(t_{\mathrm{R}},{\mathbf{r}}_{\mathrm{R}}) \in {\Bbb R}^4$ in some inertial reference frame ${\cal{I}}$ at which it is generated. A measurement event ${\cal{M}}_{X,A}$ is thus modeled as an input SRV *X* together with an output SRV *A*. As in typical studies of Bell experiments, here we consider the measurement process as instantaneous, i.e., *X* and *A* share the same space–time coordinates. Denote a causal order relation between two SRV’s *X* and *A* by *X* → *A* if *t*_*X*_ < *t*_*A*_ in all inertial reference frames, i.e., *A* is in the future light cone of *X* (so that *X* may cause *A*). A pair (*A*_*j*_, *A*_*k*_) of SRVs is space-like separated if $\Delta s^2: = |{\mathbf{r}}_{A_j} - {\mathbf{r}}_{A_k}|^2 - c^2(t_{A_j} - t_{A_k})^2 > 0$.

We will have an occasion to distinguish the specific space–time location at which correlations between random variables manifest themselves, i.e., the particular space–time location at which the correlations are registered, from the space–time locations at which the random variables themselves are generated. Accordingly, we label by ${\cal{C}}_{A_j,A_k}$ the SRV denoting the correlations between the output SRVs *A*_*j*_ and *A*_*k*_ with its associated space–time location $(t_{{\cal{C}}_{A_j,A_k}},{\mathbf{r}}_{{\cal{C}}_{A_j,A_k}})$ being at the earliest (smallest *t*) intersection of the future light cones of *A*_*j*_ and *A*_*k*_ in the reference frame ${\cal{I}}$. The general multiparty no-signaling constraints are usually stated as follows (see for example, ref. ^19^):

2 $$\begin{array}{l}\mathop {\sum}\limits_{a_j} {P_{A_1, \ldots ,A_n|X_1, \ldots ,X_n}} (a_1, \ldots ,a_j, \ldots ,a_n|x_1, \ldots ,x_j, \ldots ,x_n) = \\ \mathop {\sum}\limits_{a_j} {P_{A_1, \ldots ,A_n|X_1, \ldots ,X_n}} (a_1, \ldots ,a_j, \ldots ,a_n|x_1, \ldots ,x{\prime}_j, \ldots ,x_n)\\ \qquad \forall j \in [n],\{ a_1, \ldots ,a_n\} \setminus a_j,\{ x_1, \ldots ,x_j,x{\prime}_j, \ldots ,x_n\} \end{array}$$

In words, the above constraints state that the outcome distribution of any subset of parties is independent of the inputs of the complementary set of parties (while Eq. (2) imposes this for subsets of *n* − 1 parties, one can straightforwardly show that this also implies that the marginal distribution for smaller-sized subsets is well-defined^19^).

### Relativistic causality

We consider the causal structure of measurement events occurring at fixed space–time locations (*t*, *r*). Within this regime, the relativistic causality constraint we consider states that

- No causal loops occur, where a causal loop is a sequence of events, in which one event is among the causes of another event, which in turn is among the causes of the first event.

Causality implies that for two causally related events taking place at two spatially separated points, the cause always occurs before the effect, and this sequence cannot be changed by any choice of a frame of reference. In other words, the correlations should be such that no local observer is able to use them to send a message superluminally to any point in space–time.

An argument detailed in Supplementary Note 4 shows that if we have only two parties A and B, then this requirement is fully equivalent to the original NS conditions. In particular, suppose one of the NS conditions is violated in the form of B’s output distribution depending on A’s input. Since by assumption A chooses her measurement freely, it must be that a superluminal signal travels from A to B informing him of her input. By considering two other observers C and D in an inertial frame moving at speed *v* with respect to A and B’s reference frame, with D’s worldline intersecting that of B, and with D using the same superluminal mechanism to inform C about A’s input, one can construct a closed causal loop of events in which A is informed about her input before she has freely made its choice (see the detailed derivation in Supplementary Note 4). In other words, the two-party NS conditions are both necessary and sufficient to ensure causality.

**Fig. 1:**
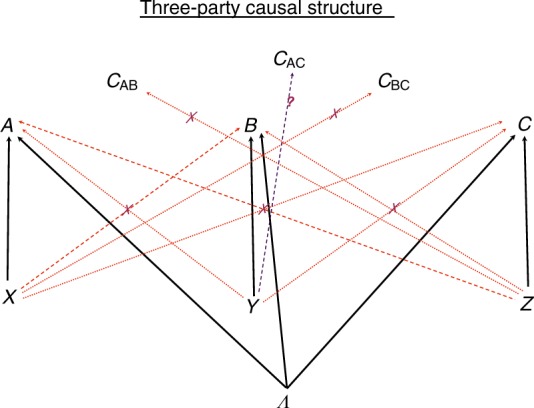
The causal structure of the three-party Bell experiment. The outputs *A*, *B*, *C* are correlated via a common Λ. The inputs *X*, *Y*, *Z* are chosen freely according to the notion of free choice. The input *X* cannot signal to change the distribution of a remote party’s output *B* or *C*, analogously for inputs *Y* and *Z*. On the other hand, depending upon the space–time configuration of the measurement events, the input may influence the correlations between remote parties’ outputs without violating causality. In particular, in the measurement configuration shown in Fig. 2, the input *Y* may influence the correlations ${\cal{C}}_{{\mathrm{AC}}}$

**Fig. 2:**
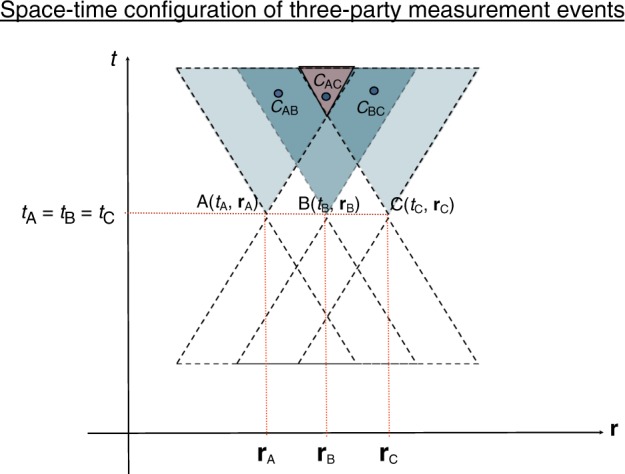
A particular space–time configuration of measurement events in the three-party Bell experiment. The space–time locations of Alice, Bob, and Charlie’s measurement events in some inertial reference frame ${\cal{I}}$ labeled by axes (*t*, **r**) are given by (*t*_A_, **r**_A_), (*t*_B_, **r**_B_), and (*t*_C_, **r**_C_), respectively. The correlations between the outputs of Alice and Bob are denoted by a space–time random variable ${\cal{C}}_{{\mathrm{AB}}}$ which manifests itself (is checked by Alice and Bob) at a space–time location within the intersection of the future light cones of Alice and Bob. Similarly, the correlations between the outputs of the other pairs of parties are denoted by ${\cal{C}}_{{\mathrm{AC}}}$ and ${\cal{C}}_{{\mathrm{BC}}}$. The crucial property of this measurement configuration is that the intersection of the future light cones of *A* and *C* is contained within the future light cone of *B*

Let us now go one step further and ask about three-party correlations. The causal structure of the three-party Bell experiment is shown in Fig. 1. Alice’s space–time random variables corresponding to her input *X* and output *A* are generated at space–time location (*t*_A_, **r**_A_) in inertial reference frame ${\cal{I}}$; similarly Bob’s input–output *Y*, *B* are generated at (*t*_B_, **r**_B_) and Charlie’s input–output *Z*, *C* are generated at (*t*_C_, **r**_C_). Consider the specific scenario in Fig. 2. The correlations between, say, A and C are represented as a random variable at a point where they are checked by the two observers when they meet, this event must take place in the intersection of A and C’s future light cones. The fundamental idea is that, as opposed to the correlations A–B and B–C, no matter where Alice and Charlie meet, they will always meet at a space–time location in the future cone of B. This means that even an instantaneous alteration of the correlations A–C by changing a setting at B’s location will be registered by the observers at a meeting point that is in the future light cone of B. So that any such action of Bob does not cause FTL signaling. This intuition is summarized and rigorously proven in the following Proposition whose proof is shown in Supplementary Note 5.

#### Proposition 1

Consider the three-party measurement configuration shown in Fig. 2, where in an inertial frame ${\cal{I}}$, the space–time locations of the measurement events (*t*_A_, **r**_A_), (*t*_B_, **r**_B_), and (*t*_C_, **r**_C_) are such that the intersection of the future light cones of *A* and *C* is contained within the future light cone of *B*. The necessary and sufficient constraints to ensure that no causality violation occurs in this configuration are given by

3 $$\begin{array}{l}P_{B,C|Y,Z}(b,c|y,z) = \mathop {\sum}\limits_a {P_{A,B,C|X,Y,Z}} (a,b,c|x,y,z) = \\ \mathop {\sum}\limits_a {P_{A,B,C|X,Y,Z}} (a,b,c|x{\prime},y,z)\;\;\;\forall x,x{\prime},y,z,b,c\\ P_{A,B|X,Y}(a,b|x,y) = \mathop {\sum}\limits_c {P_{A,B,C|X,Y,Z}} (a,b,c|x,y,z) = \\ \mathop {\sum}\limits_c {P_{A,B,C|X,Y,Z}} (a,b,c|x,y,z{\prime})\;\;\;\forall z,z{\prime},x,y,a,b\\ P_{A|X}(a|x) = \mathop {\sum}\limits_{b,c} {P_{A,B,C|X,Y,Z}} (a,b,c|x,y,z) = \\ \mathop {\sum}\limits_{b,c} {P_{A,B,C|X,Y,Z}} (a,b,c|x,y{\prime},z{\prime})\;\;\;\forall y,y{\prime},z,z{\prime},x,a\\ P_{C|Z}(c|z) = \mathop {\sum}\limits_{a,b} {P_{A,B,C|X,Y,Z}} (a,b,c|x,y,z) = \\ \mathop {\sum}\limits_{a,b} {P_{A,B,C|X,Y,Z}} (a,b,c|x{\prime},y{\prime},z)\;\;\;\forall x,x{\prime},y,y{\prime},z,c.\end{array}$$

We may fix the space–time coordinates (*t*_A_, **r**_A_) and (*t*_C_, **r**_C_) of Alice and Charlie, and investigate from which space–time locations (*t*_B_, **r**_B_) Bob is able to influence the correlations *P*_*A*,*C*|*X*,*Z*,*Y*_(*a*,*c*|*x*,*z*,*y*) without affecting the marginal distributions *P*_*A*|*X*_ and *P*_*C*|*Z*_, i.e., in which measurement configurations (3) is necessary and sufficient to prevent the violation of causality, this is done in Supplementary Note 5. The above proposition also has an immediate generalization to the general *n*-party scenario for *n* ≥ 3, this is shown in Supplementary Note 6.

### Free choice in Bell experiments

Before asking about the possible information-theoretic consequences of the modification from no signaling to relativistic causality constraints, let us ask about the possible implications for the assumption of “free-will” on the measurement settings in a Bell experiment. It is known that the definition of free will in an *n*-party Bell experiment is of the form (formulated originally by Bell in ref. ^26^)

#### Lemma 2

(Free-will notion in refs. ^10,12^) Consider the *n*-party Bell experiment, where the state of the system is denoted by Λ, the input and output of the *i*th party by *X*_*i*_, *A*_*i*_ for *i* ∈ [*n*]. Then *X*_*i*_ is free if the following condition is satisfied:

4 $$P_{X_i|{\mathbf{X}} \setminus X_i,{\mathbf{A}} \setminus A_i,\Lambda }(x_i|{\mathbf{x}} \setminus x_i,{\mathbf{a}} \setminus a_i,\lambda ) = P_{X_i}(x_i).$$

The above definition means something very natural, namely that the choice of the settings is free if and only if it is only correlated with events that take place in its future. This means, in other words, that nobody in the universe could predict the position of the setting better than randomly. In view of our previous considerations, one must extend the definition: indeed for the multiparty situation, the setting of Bob does not need to be independent on the correlations ${\cal{C}}_{{\mathrm{AC}}}$ even if both individual events that contribute to the correlation happen outside the future of B. This leads to the following modification:

#### Lemma 3

(Modified notion of free will) Consider the *n*-party Bell experiment, where the state of the system is denoted by Λ, the input and output of the *i*th party by *X*_*i*_, *A*_*i*_ for *i* ∈ [*n*]. Let ${\mathbf{A}}_{X_i \nrightarrow}=\{A_j\}$ denote a set of outputs *A*_*j*_ such that the correlation SRV $C_{\{ A_j\} }$ between all the outputs *A*_*j*_ is generated outside the future light cone of *X*_*i*_. Then *X*_*i*_ is free if the following condition is satisfied:

5 $$P_{X_i|{\mathbf{X}} \setminus X_i,{\mathbf{A}}_{X_i \nrightarrow},\Lambda }(x_i|{\mathbf{x}} \setminus x_i,{\mathbf{a}}_{x_i \nrightarrow },\lambda ) = P_{X_i}(x_i).$$

This new definition of free will and its duality with the relativistic causality constraints is elaborated more in Supplementary Note 9.

### Device-independent cryptography against relativistic adversaries

The considerations of the previous sections have important implications for post-quantum cryptographic tasks in the device-independent scenario, where the honest parties are not assumed to know the exact internal workings of their device and the eavesdropper is only constrained by the laws of relativity, see for example, refs. ^6,7,31^. In particular, two important considerations appear.

1. First, note that the set of boxes obeying the relativistic causality considerations forms a larger-dimensional polytope than the usual no-signaling polytope. This confers a larger set of attack strategies for an eavesdropper who may prepare boxes for the honest parties from this larger set. As shown in Supplementary Note 11, in certain known device-independent protocols for the cryptographic task of randomness amplification^11,18^, this larger set of attack strategies can severely compromise the security of the protocol, if the honest parties were to perform the required Bell test in a space–time measurement configuration where the superluminal influence can take effect.
2. Second, and crucially, the security of cryptographic protocols (even relying on a two-party Bell test) can be compromised when the measurement event of Eve’s system happens in a suitable space–time location. In particular, as shown in Supplementary Note 14, the property of monogamy of nonlocal correlations can break down under the relativistic causality constraints so that such an eavesdropper can obtain full information about the output of the honest parties in the protocol.

Remark that the first type of attack strategy above may be circumvented if the honest parties perform their measurements in a carefully chosen measurement configuration where the usual no-signaling constraints (2) are both necessary and sufficient. In contrast, the second type of attack can only be avoided if certain assumptions are made about the space–time location of the eavesdropper’s measurement event, or alternatively if the honest parties’ systems are assumed to be sufficiently shielded from all influences, even those respecting causality.

### *v*-Causal models and genuine multiparty nonlocality

In view of the consequences to free will, and device-independent cryptography and randomness generation as well as the breakdown of monogamy of nonlocal correlation, one may expect that a recent milestone result concerning the refutation of the so-called *v*-causal models^27,32^ also does not hold under the new paradigm. Indeed, if we take the measurement configurations of refs. ^27,32^, a straightforward analysis reveals that these results do not hold under the modified free-choice definition in Lemma 3. However, we found a new measurement configuration and a modification of the analysis (shown in detail in Supplementary Note 15) that still allows for refutation of *v*-causal models under the true relativistic causality and free-will requirements, modulo the assumptions of ref. ^27^.

The previous considerations also are highly significant in formulating a precise notion of genuine multiparty nonlocality^33,34^. Here we revisit the notion of a genuine three-way nonlocality, as pioneered by Svetlichny^33–35^ in light of the considerations of the previous sections. In particular, we introduce the notion of relativistically causal bilocal (RCBL) correlations and propose an inequality of the violation, which suggests that the resulting correlations *P*_*A*,*B*,*C*|*X*,*Y*,*Z*_ are genuinely relativistically causal three-way nonlocal.

#### Definition 4

Suppose that *P*_*A*,*B*,*C*|*X*,*Y*,*Z*_(*a*,*b*,*c*|*x*,*y*,*z*) can be written in the form

6 $$\begin{array}{l}P_{A,B,C|X,Y,Z}(a,b,c|x,y,z) = \\ r_{AB|C}\mathop {\sum}\limits_\lambda {q_\Lambda } (\lambda )P_{A,B|X,Y,\lambda }(a,b|x,y)P_{C|Z,\lambda }(c|z)\\ + r_{AC|B}\mathop {\sum}\limits_\gamma {q_\Gamma } (\gamma )P_{A,C|X,Y,Z,\Gamma }(a,c|x,y,z,\gamma )P_{B|Y,\Gamma }(b|y,\gamma )\\ + r_{BC|A}\mathop {\sum}\limits_\upsilon {q_\Upsilon } (\upsilon )P_{B,C|Y,Z,\Upsilon }(b,c|y,z,\upsilon )P_{A|X,\Upsilon }(a|x,\upsilon )\end{array}$$

with *r*_*AB*|*C*_, *r*_*AC*|*B*_, *r*_*BC*|*A*_ ≥ 0, *r*_*AB*|*C*_ + *r*_*AC*|*B*_ + *r*_*BC*|*A*_ = 1, and $\mathop {\sum}\limits_\lambda {q_\Lambda } (\lambda ) = \mathop {\sum}\limits_\gamma {q_\Gamma } (\gamma ) = \mathop {\sum}\limits_\upsilon {q_\Upsilon } (\upsilon ) = 1$, where the terms obey the relativistic causality constraints in Eq. (3), i.e., each of the marginals *P*_*A*|*X*_, *P*_*B*|*Y*_, and *P*_*C*|*Z*_ is well-defined independently of the other parties’ inputs, while the two-party term *P*_*A*,*C*|*X*,*Y*,*Z*,Γ_ exhibits an explicit dependence on *Y*. Then the correlations *P*_*A*,*B*,*C*|*X*,*Y*,*Z*_(*a*,*b*,*c*|*x*,*y*,*z*) are said to be relativistically causal bilocal (RCBL). Otherwise, we say that they are genuinely tripartite relativistically causal nonlocal.

We compare the set of RCBL correlations with the existing notions of a genuine multiparty nonlocality^33,34^, namely bilocal (BL) correlations and no-signaling bilocal (NSBL) correlations, showing that these notions are inequivalent. Quite intriguingly, we find a new aspect of the set of quantum correlations (Q), namely that it is not contained within the set of relativistic causal bilocal correlations, showing a stronger version of a genuine multiparty nonlocality of quantum correlations than those considered so far^36^.

In particular, we prove the following Lemma 5 in Supplementary Note 16.

#### Lemma 5

Consider the three-party Bell scenario, with each party performing one of two dichotomic measurements. In measurement configurations such as Fig. 2, the following inequality holds for all relativistically causal bilocal boxes *P*_*A*,*B*,*C*|*X*,*Y*,*Z*_ ∈ *RCBL*.

7 $$\begin{array}{l}{\cal{I}}_{{\mathrm{RCBL}}}: = 2\langle A_1B_1\rangle + \langle A_1C_1\rangle _{y = 1} + \langle A_1C_1\rangle _{y = 2} + 2\langle B_1C_2\rangle \\ - 2\langle A_2B_2C_1\rangle + 2\langle A_2B_2C_2\rangle \le 6.\end{array}$$

Suitably chosen measurements on the *GHZ* state $|GHZ\rangle = \frac{1}{{\sqrt 2 }}\left( {|000\rangle + |111\rangle } \right)$ attain a value $2(1 + 2\sqrt 2 ) \approx 7.657$ violating the inequality, showing that quantum correlations are genuinely tripartite relativistically causal nonlocal. Furthermore, these quantum correlations belong to the set *BL*, hence $BL \not\subseteq RCBL$.

The above result gains additional importance in light of recent investigations into the causal hierarchy of multiparty correlations^36^, even going beyond Bell’s theorem^37^.

## Discussion

In this paper, we have examined the no-signaling constraints from the point of view of causality and proposed a modification to these constraints to a subset that ensures preservation of causality depending on the space–time configuration of measurement events. As a consequence, we find that boxes in the post-quantum scenario, where one only restricts to the constraints imposed by relativistic causality, should be labeled by the space–time locations of the parties performing the measurement. The correlations that the parties observe can then exhibit various dependencies while still being consistent with relativistic causality.

Relativistic causality, if strictly applied, gives much more freedom for correlations than the no-signaling conditions. The latter were just inherited from quantum mechanics and certainly make sense if the only carriers of physical interactions are local fields that manifest themselves as particles. This is the case in the Standard Model; however, it is not known whether there are such models in the gravitational fields (among others due to the problem of renormalization). The appearance of the new type of mediating field that is manifestly nonlocal, i.e., does not carry information from one point to another, but from a point to a space–time region might be the signature that there is a chance for a footbridge between gravity and quanta. To make the picture nontrivial, it is necessary that the field be faster than light. Its possible nonlocal character is consistent with the conclusions that were obtained about tachyons that cannot be localized^38,39^. Another important element in support of the above ideas is Rudolf Haag’s observation in the classic text “Local Quantum Physics”^40^ that the correspondence between the particle and the field holds only in the asymptotic regime of infinite time, where the free fields obey the dynamics and commutation equations so as to describe the localized particles. In other words, it may well be that the exact quantum field theory does not describe particles, which may be only our model idealizations.

## Supplementary information

Supplementary Information

## Data Availability

Data sharing not applicable to this article as no datasets were generated or analyzed during the current study.

## References

[1] Einstein A, Podolsky B, Rosen N (1935). Can quantum mechanical description of physical reality be considered complete?. Phys. Rev..

[2] Bell JS (1964). On the Einstein-Podolsky-Rosen paradox. Physics.

[3] Hensen B (2015). Loophole-free Bell inequality violation using electron spins separated by 1.3 kilometres. Nature.

[4] Giustina M (2015). A significant-loophole-free test of Bell’s theorem with entangled photons. Phys. Rev. Lett..

[5] Shalm LK (2015). A strong loophole-free test of local realism. Phys. Rev. Lett..

[6] Barrett J, Hardy L, Kent A (2005). No signalling and quantum key distribution. Phys. Rev. Lett..

[7] Barrett J, Colbeck R, Kent A (2012). Unconditionally secure device-independent quantum key distribution with only two devices. Phys. Rev. A..

[8] Pironio S (2010). Random numbers certified by Bell’s theorem. Nature.

[9] Buhrman H, Cleve R, Massar S, de Wolf R (2010). Non-locality and communication complexity. Rev. Mod. Phys..

[10] Colbeck R, Renner R (2012). Free randomness can be amplified. Nat. Phys..

[11] Brandão FGSL (2016). Realistic noise-tolerant randomness amplification using finite number of devices. Nat. Commun..

[12] Colbeck R, Renner R (2011). No extension of quantum theory can have improved predictive power. Nat. Commun..

[13] Popescu S, Rohrlich D (1994). Quantum nonlocality as an axiom. Found. Phys..

[14] Popescu, S. & Rohrlich, D. in *The Proceedings of the Symposium on Causality and Locality in Modern Physics and Astronomy: Open Questions and Possible Solutions*. (York University, Toronto, 1997).

[15] Pawłowski M (2009). Information causality as a physical principle. Nature.

[16] Brassard G (2006). Limit on nonlocality in any world in which communication complexity is not trivial. Phys. Rev. Lett..

[17] Ramanathan R (2016). Randomness amplification against no-signaling adversaries using two devices. Phys. Rev. Lett..

[18] Gallego R (2013). Full randomness from arbitrarily deterministic events. Nat. Commun..

[19] Masanes L, Acin A, Gisin N (2006). General properties of nonsignaling theories. Phys. Rev. A..

[20] Bohm, D. & Hiley, B. J. *The Undivided Universe: An Ontological Interpretation of Quantum Theory* (Routledge, UK, 1995).

[21] Eckstein, M. & Miller, T. Causality for nonlocal phenomena, arXiv:1510.06386 (2015).

[22] Grunhaus J, Popescu S, Rohrlich D (1996). Jamming nonlocal quantum correlations. Phys. Rev. A..

[23] Shimony, A. in *Foundations of Quantum Mechanics in Light of the New Technology* (eds Kamefuchi, S. et al.) (Japan Physical Society, Tokyo, 1983).

[24] Toner B (2009). Monogamy of non-local quantum correlations. Proc. R. Soc. A.

[25] Colbeck, R. & Renner, R. A short note on the concept of free choice, arXiv:1302.4446 (2013).

[26] Bell, J. S. in *Speakable and Unspeakable in Quantum Mechanics*, Ch. 12 (Cambridge University Press, UK, 1987).

[27] Bancal JD (2012). Quantum nonlocality based on finite-speed causal influences leads to superluminal signaling. Nat. Phys..

[28] Eberhard PH, Ross RR (1989). Quantum field theory cannot provide faster-than-light communication. Found. Phys. Lett..

[29] Weinberg S (1989). Testing quantum mechanics. Ann. Phys..

[30] Gisin N (1990). Weinberg’s non-linear quantum mechanics and supraluminal communications. Phys. Lett. A.

[31] Barrett J, Kent A, Pironio S (2006). Maximally non-local and monogamous quantum correlations. Phys. Rev. Lett..

[32] Barnea TJ, Bancal JD, Liang YC, Gisin N (2013). Tripartite quantum state violating the hidden influence constraints. Phys. Rev. A..

[33] Bancal JD, Barrett J, Gisin N, Pironio S (2013). The definition of multipartite nonlocality. Phys. Rev. A..

[34] Gallego R, Würflinger LE, Acín A, Navascués M (2012). An operational framework for nonlocality. Phys. Rev. Lett..

[35] Svetlichny G (1987). Distinguishing three-body from two-body nonseparability by a Bell-type inequality. Phys. Rev. D..

[36] Chaves R, Cavalcanti D, Aolita L (2017). Causal hierarchy of multipartite Bell nonlocality. Quantum.

[37] Fritz T (2016). Beyond Bell’s Theorem II: Scenarios with arbitrary causal structure. Comm. Math. Phys..

[38] Bers, A., Fox, R., Kuper, C. G. & Lipson, S. G. in *Relativity and Gravitation* (eds Kuper, C. G. & Peres, A.) 41 (Gordon and Breach Science Publishers, New York, 1971).

[39] Fox R, Kuper CG, Lipson SG (1969). Do faster-than-light group velocities imply violation of causality?. Nature.

[40] Haag R (1992). Local Quantum Physics.

